# Successful reversal of remimazolam anesthesia in a “cannot intubate, can ventilate” situation: a case report

**DOI:** 10.1186/s40981-023-00638-4

**Published:** 2023-07-24

**Authors:** Shota Sekimoto, Shuya Kiyama, Shoichi Uezono

**Affiliations:** Department of Anesthesiology, School of Medicine, The Jikei University, Nishi-Shimbashi 3-25-8, Minato-Ku, Tokyo, 105-8461 Japan

**Keywords:** Remimazolam, Flumazenil, Epiglottis, Difficult laryngoscopy, CICV

## Abstract

**Background:**

Compared to other intravenous anesthetics, availability of a specific antagonist flumazenil is a clear advantage of remimazolam. We report a patient who could be rapidly woken up when laryngoscopy and tracheal intubation were unexpectedly difficult.

**Case presentation:**

A 62-year-old man was scheduled to have resection of a small gingival tumor. Preoperative airway examination was unremarkable except for an omega-shaped epiglottis. Anesthesia was induced with remifentanil/remimazolam infusion and rocuronium. A small omega-shaped edematous epiglottis precluded identification of glottis. Consciousness and spontaneous ventilation were rapidly restored after administration of flumazenil and sugammadex. Tracheostomy was done under local anesthesia while the patient breathed spontaneously.

**Conclusions:**

Remimazolam can be a reasonable induction agent when there are concerns regarding airway management. Avoiding repeated airway manipulations is extremely important to prevent deterioration into a “cannot intubate, cannot ventilate (CICV)” emergency.

## Background

Remimazolam, a short-acting benzodiazepine, is unique in that it has a specific antagonist flumazenil, compared to other commonly used induction agents such as propofol or thiopental. Use of flumazenil after remimazolam anesthesia has been reported when recovery of consciousness was unduly delayed [1]. Theoretically, reversal of remimazolam effect by flumazenil may also help prevent “cannot intubate, cannot ventilate (CICV)” in case of unexpected difficult airway [2]; however, such cases have not been reported under impending CICV situation. We wish to report a patient in whom flumazenil was used to rapidly restore consciousness and spontaneous ventilation after unexpectedly difficult laryngoscopy. We discuss potential role of remimazolam in the context of a difficult airway.

## Case presentation

**Fig. 1:**
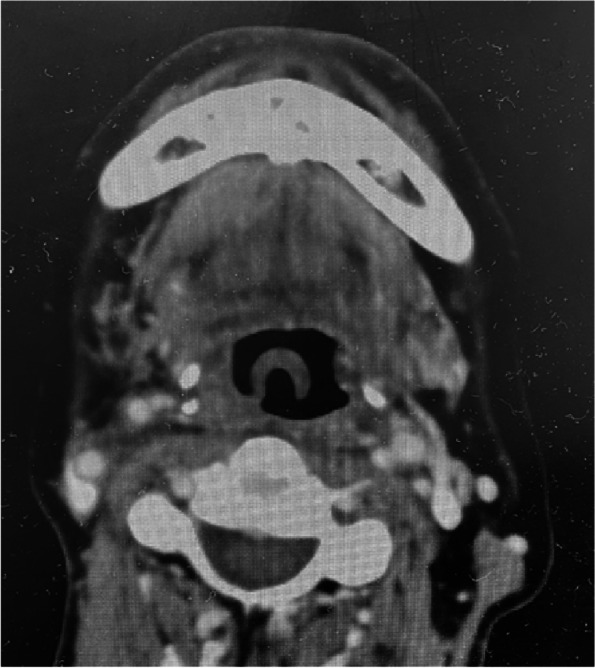
A computed tomography done 2 weeks before surgery. An omega-shaped epiglottis shown in a CT scan

**Fig. 2:**
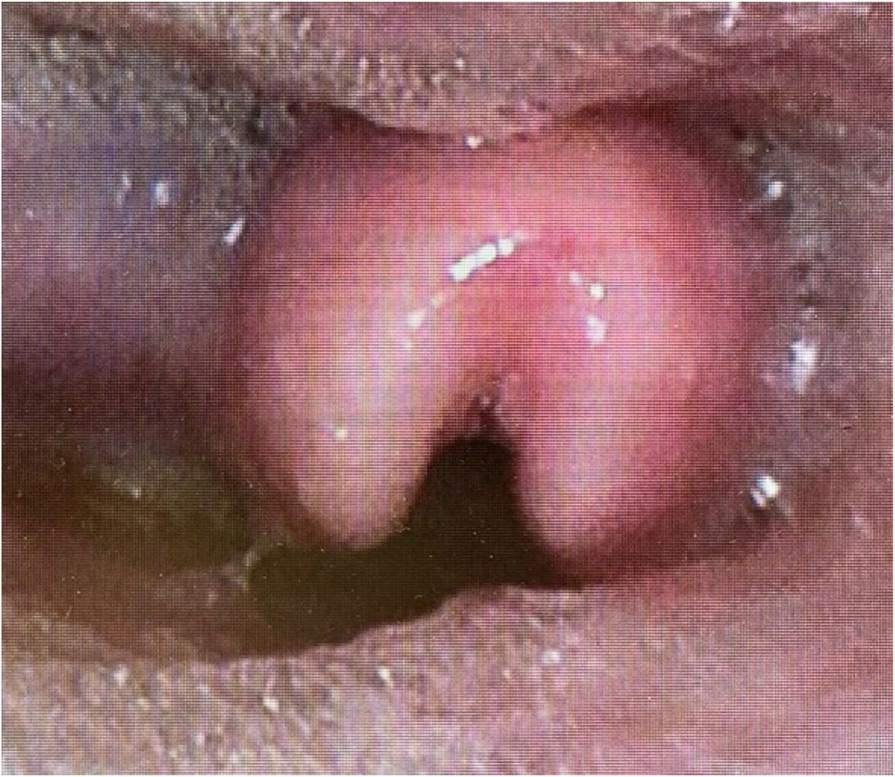
Fiberoptic examination finding at the outpatient clinic. An omega-shaped epiglottis was detected by fiberoptic examination, but the vocal cords were not visible

Written informed consent for publication was obtained from the patient reported here. A 62-year-old man was scheduled for resection of a small malignant tumor (size 1 cm) of his right lower gingiva. His height and weight were 168 cm and 62 kg, respectively, with body mass index of 22.0 kg/m^2^. Ear, nose, and throat (ENT) surgeons requested nasal intubation to have a good surgical field. The patient had a history of resection of hypopharyngeal cancer 14 years ago. At that time, tracheostomy was done, but the tracheal stoma had been later closed uneventfully. He also had postoperative radiation therapy to his neck. Information about prior anesthetic and airway management were not available. On the day before elective surgery for the gingival tumor, the patient did not have any symptoms suggesting a narrowed upper airway. Specifically, he was not dyspneic in supine position and had neither hoarseness nor dysphagia. He could open his mouth more than 3 fingerbreadths, and Mallampati score was 1. Neck movement was not limited. A CT scan of head and neck which was done 2 weeks before this surgery showed an omega-shaped epiglottis (Fig. 1). Fiberoptic examination done at ENT outpatient clinic at the same time also confirmed the shape of epiglottis (Fig. 2). Both nasal cavities were patent, and the trachea was not deviated. Based on these physical and radiological findings, we did not consider awake tracheal intubation was indicated for this patient. However, we were concerned about the omega-shaped epiglottis, which may make laryngoscopy difficult. Therefore, we planned anesthesia induction using remimazolam and remifentanil, as effects of these drugs can be antagonized or rapidly wear off if necessary.

After application of standard monitoring (ECG, non-invasive blood pressure, pulse oximetry, capnography, neuromuscular transmission, and bispectral index), 75 μg of fentanyl was given followed by remifentanil infusion at a rate of 0.1 μg/kg/min, while the patient breathed 100% oxygen (6 l/min) from a face mask. Remimazolam was infused at a rate of 150 mg/h (approximately 2.4 mg/kg/h), reduced to 60 mg/h after loss of response to calling the patient’s name. Sixty milligrams of rocuronium was given, and mask ventilation was started. Normal capnogram of rectangular shape was easily obtained. After preparation of his left nasal cavity by cotton swabs soaked in lidocaine/adrenaline, a single-use fiberoptic bronchoscope (Ambu® aScope 4™ Broncho, Ambu A/S, Baltorpbakken, Denmark) was nasally inserted. An assistant placed a video laryngoscope (McGRATH™ MAC, Medtronic, Boulder, CO, USA) into oral cavity. An omega-shaped, edematous epiglottis was easily visualized, but neither the vocal cords nor glottic opening was identified. Despite three attempts, we could not locate the glottis. Although the shape of the capnogram was still rectangular and oxygen saturation was maintained above 97%, further airway instrumentation was abandoned, and we decided to wake the patient up and restore spontaneous ventilation. The ENT surgeons also watched a view of the swollen epiglottis and agreed to perform tracheostomy after the patient regained consciousness. Infusion of remifentanil and remimazolam was terminated, followed by injection of 200 mg of sugammadex and 0.5 mg of flumazenil. Train-of-four (TOF) measured every 12 s was fluctuating between 0 and 1 immediately before giving sugammadex. Three minutes after administration of sugammadex and flumazenil, the patient opened his eyes, and TOF returned to more than 100%. Almost at the same time of eye opening, spontaneous respiration was restored with a rate of 10/min and end-tidal CO_2_ of 50 mmHg. The patient could understand our explanation about the situation and necessity of front-of-neck airway. Tracheostomy was done under local anesthesia, and a 7.0-mm reinforced tracheal tube was placed. Once correct placement of the tube was confirmed by chest auscultation and capnography, anesthesia was induced again with propofol and maintained with sevoflurane. Subsequent tumor resection and postoperative course was uneventful. Tracheostomy was closed 4 days later.

## Discussion

Choice of induction and securing airway is critical in patients with a potentially difficult airway. The most important decision is whether to intubate while the patient is awake or after induction of anesthesia. Many airway guidelines emphasize this [3, 4], but specific induction agents are not recommended. Hirota has recently suggested that remimazolam could be a reasonable choice to prevent CICV, as its effect can be antagonized by flumazenil [2].

Except for previous surgery and radiotherapy for hypopharyngeal cancer, the patient did not have any symptoms or signs which suggest difficult intubation. The gingival tumor was small and existed on the right lateral side of oral cavity. We therefore did not judge that the tumor itself would make laryngoscopy or intubation difficult. Our only concern was the shape of epiglottis detected on preoperative CT and laryngoscopy. It is sometimes difficult to lift an omega-shaped epiglottis, which looks like that of an infant, in order to visualize glottic opening by either direct or video laryngoscopes. Veiga et al. reported a middle-aged man with worsening dyspnea presented for microlaryngeal surgery, in whom an omega-shaped epiglottis was found at videolaryngoscopy and a 4.0-mm cuffed microlaryngeal tube was placed with some difficulty [5]. Although an omega-shaped epiglottis itself is not necessarily pathological, it may be associated with chronic inflammation of epiglottis [6].

At videolaryngoscopy using McGRATH™ MAC, the shape of epiglottis was the same as we had imagined. However, unexpected findings were the edematous epiglottis and surrounding tissue. Three attempts of fiberoptic laryngoscopy failed to locate the vocal cords and glottis. At this point, mask ventilation was still possible, but we decided to wake the patient up rather than to proceed to alternative airway manipulations, such as placement of a supraglottic airway. Airway management guideline of the Japanese Society of Anesthesiologists (JSA) recommends to consider restoring consciousness and spontaneous ventilation in the Yellow Zone (failed tracheal intubation). Naguib and colleagues performed simulations to predict the duration of unresponsiveness and ventilatory depression in a common induction using fentanyl, propofol, and neuromuscular blocking agent [7]. They report that despite the full reversal of rocuronium-induced neuromuscular blockade by a large dose of sugammadex, intolerable ventilatory depression persists particularly in obese patients. Curtis et al. reports an elderly patient with tongue base cancer, who deteriorated into CICV situation after propofol/rocuronium induction and multiple laryngoscopy attempts. Emergency administration of 15 mg/kg of sugammadex fully restored the neuromuscular function, but not the upper airway patency [8]. These reports highlight that both consciousness and normal muscular function are essential to maintain airway patency.

Although the dose of sugammadex (approximately 3 mg/kg) was much less than recommended dose of 16 mg/kg for emergency reversal, 22 min had already elapsed after the administration of rocuronium; therefore, we gave 200 mg as one vial of sugammadex was readily available on the anesthesia cart. The TOF ratio rapidly increased to 100% after this relatively small dose. When the patient started to breathe spontaneously, effect-site remifentanil concentration was predicted to be 1.0 ng/mL based on a pharmacokinetic model of Minto et al. [9], which is compatible with spontaneous respiration.

Another issue to be discussed is the dose of flumazenil to rapidly restore consciousness. When flumazenil is used to expedite recovery from remimazolam anesthesia, it is recommended to give a dose of 0.2 mg slowly. It may be argued that the dose of 0.5 mg given to this patient was excessive. However, it is unknown how much flumazenil should be given in impending or true CICV emergency. As we wanted to restore the patient’s consciousness as quickly and fully as possible, we gave him a dose of 0.5 mg, and no adverse effects such as hypertension, tachycardia, and convulsion were observed. We emphasize that the dose should be decided in each clinical circumstance, particularly in patients who are long-term benzodiazepine users.

Our patient was fortunately not in a state of CICV, but he was definitely “cannot intubate, but still can ventilate” and, in other words, in the Yellow Zone of JSA airway management guideline. Repeated attempts of laryngoscopy and intubation could have worsened airway edema and caused bleeding. Early decision to stop remimazolam/remifentanil infusion and pharmacological reversal by flumazenil and sugammadex avoided deterioration into a CICV crisis. If we had induced anesthesia using other intravenous agents instead of remimazolam, it could have taken longer to wake the patient up and restore spontaneous ventilation. Although remimazolam induction per se does not necessarily guarantee safe airway management, remimazolam can be a reasonable agent in a patient with a potentially difficult airway. Finally, careful planning regarding methods of securing an airway and anesthesia induction is always important when concern of airway status exists.

## Data Availability

The data used in this report are available from the corresponding author upon reasonable request.

## Abbreviations

CICV: Cannot intubate, cannot ventilate
ENT: Ear, nose, and throat
JSA: Japanese Society of Anesthesiologists
TOF: Train-of-four
